# Application of Microarray-Based Comparative Genomic Hybridization in Prenatal and Postnatal Settings: Three Case Reports

**DOI:** 10.4061/2011/976398

**Published:** 2011-08-07

**Authors:** Jing Liu, Francois Bernier, Julie Lauzon, R. Brian Lowry, Judy Chernos

**Affiliations:** Department of Medical Genetics, University of Calgary, 2888 Shaganappi Trail NW, Calgary, AB, T3B 6A8, Canada

## Abstract

Microarray-based comparative genomic hybridization (array CGH) is a newly emerged molecular cytogenetic technique for rapid evaluation of the entire genome with sub-megabase resolution. It allows for the comprehensive investigation of thousands and millions of genomic loci at once and therefore enables the efficient detection of DNA copy number variations (a.k.a, cryptic genomic imbalances). The development and the clinical application of array CGH have revolutionized the diagnostic process in patients and has provided a clue to many unidentified or unexplained diseases which are suspected to have a genetic cause. In this paper, we present three clinical cases in both prenatal and postnatal settings. Among all, array CGH played a major discovery role to reveal the cryptic and/or complex nature of chromosome arrangements. By identifying the genetic causes responsible for the clinical observation in patients, array CGH has provided accurate diagnosis and appropriate clinical management in a timely and efficient manner.

## 1. Introduction

Genomic disorders, resulting from DNA rearrangements involving region-specific repeat sequences, are caused by abnormal dosage of one or more genes located within the rearranged genomic fragments. Cytogenetic analysis has been a useful diagnostic tool for this disease category especially in idiopathic developmental delay/mental retardation, multiple congenital anomalies, dysmorphism, and pregnancy at risk for chromosomal abnormalities. However, the limitation of band resolution in the conventional cytogenetic methodology karyotype (5–10 Mb) has prompted the development of technologies which can identify previously unrecognized chromosomal anomalies. Since Solinas-Toldo et al. published the first article on array-based comparative genome hybridization (array CGH) in 1997 [1], this technique has become one of the fastest growing ones due to its ability to screen a sample for thousands to millions of different loci at once. The array CGH platforms used for clinical diagnosis are able to detect nonmosaic and mosaic aneuploidies, subtelomeric imbalances, known microdeletion/microduplication syndromes, and other unique unbalanced chromosomal rearrangements [2–4]. The detection rate has been improved to 10–16% in patients with a normal karyotype [5, 6]. In addition, array CGH is able to uncover numerous copy number variations (CNVs) of not-yet-known clinical significance scattered throughout the human genome.

In pediatric patients with idiopathic developmental delay and dysmorphic features, it is difficult to come up with a specific diagnosis due to the lack of cardinal features of a syndrome. Similarly, in the prenatal setting when congenital anomalies are seen which do not suggest a specific syndrome, it is especially difficult to make the diagnosis within a limited time frame so that appropriate management can be performed. In these cases, the use of array CGH has demonstrated great advantages to both patients and physicians. First of all, the multiplex format of array enables the simultaneous screening of hundreds of well-characterized disease loci and the subtelomeric regions, leading to the shortened diagnostic process and reduced cost compared to ordering sequential tests for each individual locus. Second, array CGH is a discovery-based approach. When a CNV with unknown clinical significance is identified, the genes located within it become the potential candidates, which facilitate further studies leading to disease gene discovery. Besides, identifying novel CNVs may also help to characterize a new genetic disorder. Third, the technical developments from arrays based on BAC (bacteria artificial chromosome) to oligonucleotide probes and the much improved array resolution enable the potential to better define the boundary of genomic gains and losses [7]. This is critical when an annotated gene is located near the boundary. The accurate definition of the genetic content within the duplicated or deleted region is essential for the appropriate clinical management of the patients and for the characterization of involved genes [8].

We have chosen to focus on patients in whom the complete cytogenetic abnormalities were principally discovered by array CGH, rather than by conventional karyotyping or fluorescence insitu hybridization. By presenting the three cases here, we would like to emphasize the power of array CGH in identifying the genetic causes responsible for the clinical presentations in the patients so that accurate diagnosis, prognosis, and clinical management can be provided and achieved.

## 2. Materials and Methods

### 2.1. Patients

The patients and their families were recruited through the Genetics Clinic at the Alberta Children's Hospital. Informed consents were obtained from the patients or their parents.

### 2.2. Array CGH

Genomic DNA was extracted from the whole blood of patients using Gentra Puregene Cell Kit (Qiagen) according to the manufacturer's protocol. Genomic DNA from each patient was labeled with red fluorescent dye cyanine-5 (Cy-5) and hybridized with same-sex normal reference DNA (Promega) labeled with green fluorescent dye Cy-3. Array CGH was performed using two platforms. The first platform, CytoChip ISCA 8 × 60 K v2.0 Oligonusleotide array (BlueGnome), consist of 60,000 oligonusleotides and evaluates the whole genome with an effective backbone resolution of 170 Kb. 137 OMIM genes are represented with average oligonucleotide spacing of 3.5 Kb and at centromeres and subtelomeres of 4.5 Kb. The second platform, Cytosurs Syndrome Plus V2 2 × 105 K Oligonucleotide array (OGT), consists of 105,000 oligonucleotides with a backbone resolution of 40 Kb. 410 genes in disease associated areas are targeted with oligonucleotide distributing every 3 Kb. Subtelomeric regions are represented with an average resolution of 40 Kb.

Hybridization of highly repetitive sequences was suppressed by addition of unlabeled *Cot-1* DNA in the reaction. Depending on the array platform used, the relative hybridization signal intensity of the patient and control was normalized using either BlueFuse Multi software v2.2 (CytoChip v2 algorithm) or Feature Extraction v10.7.3.1 (Agilent Technologies) and Cytosur 2.5.3 (OGT). The value of each probe was calculated by the log2 of the Cy5/Cy3 ratios and plotted along each chromosome at the corresponding locus. Genomic gains and losses were called by the software when the average value of at least three consecutive oligo probes is over +0.35 or below −0.4, respectively. Called imbalances were further aligned with the in-house database and the Database of Genomic Variants (DGV, NCBI 36/HG 18) to exclude benign copy number variations (CNVs). Finally, the imbalances were examined in the USCS Genome Browser (NCBI 36/HG 18) to determine whether they are causative for the patient's clinical presentation.

### 2.3. FISH Validation

The copy number variations identified by a CGH, which we considered for further validation, were analyzed using fluorescence in situ hybridization (FISH) analysis. FISH probes were either chosen from the eFISH (build 36) and ordered from the Toronto Centre for Applied Genomics or purchased commercially. FISH was performed on metaphase chromosomes or interphase nuclei using the standard FISH clinical protocol preestablished in the Cytogenetic Laboratory in the Alberta Children's Hospital. Parental blood samples were examined to determine the inheritance pattern of the variations.

## 3. Results and Discussion

### 3.1. Case No. 1

**Figure 1:**
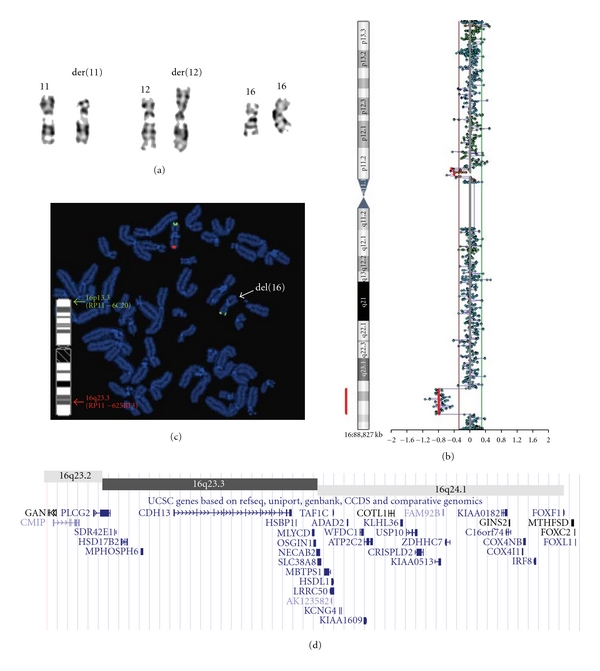
Apparently balanced translocation is accompanied by a cryptic genomic deletion (a) Conventional karyotyping revealed a reciprocal translocation involving the short arm of one chromosome 11 (11p14) and the short arm of one chromosome 12 (12p13.2), which appears to be balanced. Chromosomes 16 appear normal. (b) Array CGH study detected a significant copy number loss on chromosome 16 (q23.2-q24.1) (c) This deletion was confirmed by FISH studies using a BAC probe RP11-625B13 (red) mapped to 16q23.3 and a control probe RP11-6C20 (green) mapped to 16p13.3. (d) Genes located within the deleted region 16q23.2-q24.1 (nucleotide 79,945,820 to 85,430,304, NCBI 36/HG 18) as shown by the UCSC genome browser. These include FOX family cluster *FOXF1*, *FOXC2,* and *FOXL1*.

The couple came to our attention due to the positive first trimester screen. The woman is a healthy 25-year-old, G1P0. The first trimester nuchal translucency screen at 13 weeks was positive, and the couple decided to proceed with chorionic villus sampling. To rule out common chromosomal aneuploidies, fluorescence in situ hybridization (FISH) studies were performed using specific probes targeting chromosome 13, 18, 21, X, and Y (AneuVysion kit, Abbott Molecular Inc.), and the results were normal. The followup G-banded chromosome study revealed a reciprocal translocation involving the short arm of one chromosome 11 and the short arm of one chromosome 12 – 46,XY,t(11;12)(p14;p13.2) (Figure 1(a)), which appeared to be balanced. To determine whether this translocation was familial, chromosome studies were performed on both parents and the results were normal. Subsequent prenatal ultrasounds showed signs of a significant cardiac defect as well as mild bilateral dilatation of the renal pelvis. Followup fetal echocardiogram confirmed the complex congenital heart defect—left ventricular hypoplasia, ventricular septal defect, and mild tricuspid insufficiency. Given the presence of the *de novo* reciprocal translocation, it was suspected that a cryptic imbalance may be present. Array CGH study was therefore performed using the CytoChip ISCA 8 × 60 K v2.0 Oligonucleotide array platform (BlueGnome) and showed that there is significant copy number loss (5.48 Mb in size) on the long arm of chromosome 16 (q23.2-q24.1) from nucleotide 79,945,820 to 85,430,304 (NCBI 36/HG 18) (Figure 1(b)). This deletion was confirmed by FISH studies using a BAC probe RP11-625B13 mapped to 16q23.3 (Figure 1(c)) and was not related to the translocation breakpoints. FISH analysis was also performed on parental blood samples which showed a normal result. Of note, this large deletion on chromosome 16 is not detectable on karyotype (Figure 1(a)). 

The Database of Genomic Variants (DGV, NCBI 36/HG 18) is a useful tool to exclude benign CNVs. DGV revealed that there are no reported benign CNVs encompassing this deleted region, suggesting that it is not likely to be a benign change. This *de novo* deletion encompasses multiple genes including *FOXF1*, and two other related genes *FOXC2* and *FOXL1* (Figure 1(d)). Deletion in *FOXF1* is associated with a lethal condition named alveolar capillary dysplasia with misalignment of pulmonary veins (ACD/MPV, OMIM no. 265380). Alveolar capillary dysplasia is characterized by abnormal development of the capillary vasculature around the alveoli leading to persistent pulmonary hypertension in the early postnatal period [9]. Affected infants are usually born without any prior suspicion of lung malformation. However, most babies deteriorate rapidly, and many die of respiratory failure within the newborn period even with significant respiratory support [10]. Other features of ACD/MPV include abnormal development of the cardiovascular, gastrointestinal, genitourinary, and musculoskeletal systems [11]. A recent array CGH study demonstrated that alveolar capillary dysplasia is caused by the genomic deletion of the *FOX* gene cluster [12]. It also suggests that the cardiovascular malformation in this disorder is caused by the haploinsufficiency for the neighboring *FOXC2* and *FOXL1* genes, as a patient with a downstream deletion encompassing just *FOXC2* and *FOXL1* exhibited cardiac defects without any respiratory complications [12]. Therefore, this deletion is considered pathogenic and would explain the fetal cardiac anomalies seen on prenatal ultrasounds and fetal echocardiogram. The prognosis for this pregnancy is extremely poor with the presence of the lethal condition ACD in addition to the fetal cardiac anomalies. The previousy identified reciprocal translocation t(11;12) and this large deletion are most likely *de novo* events which occurred together within a single germ cell. Therefore, the recurrence risk will be low for future pregnancies. 

The majority of apparently balanced structural rearrangements are not associated with an abnormal phenotype. After the identification of such rearrangements, it is important to test the parents and determine whether it is inherited or *de novo*. Inherited rearrangements have reduced clinical concerns and are likely to be benign. However, *de novo* apparently balanced rearrangements predispose to a higher risk of a cryptic genomic imbalance at the breakpoints or somewhere else, which may be causative for the clinical phenotypes in the patients. Therefore, prenatal array CGH should be recommended in such circumstances for better clinical management. 

### 3.2. Case No. 2

A 2-year boy was referred to the genetics clinic for assessment of his recurrent thrombocytopenia, short stature, and dysmorphic features. He was found to have isolated thrombocytopenia during acute illnesses which would resolve spontaneously with resolution of the intercurrent illness. Platelet count was below normal when well. Hematologic investigations included a normal peripheral blood smear, bone marrow aspirate and biopsy, and a normal immune workup. His past medical history was significant for vesicoureteral reflux, hypospadias, bilateral cryptorchidism, laryngomalacia, eczema, and constipation. He has mild fine motor and speech delays as well as behavioural issues including poor socialization, aggressive behaviour, and decreased attention. On physical examination at 6 years of age his growth parameters were all below the 3rd percentile. He had coarse hair, hypertelorism, epicanthal folds, short palpebral fissures, a depressed nasal root with a bulbous nasal tip, small teeth, low-set ears which were posteriorly angulated as well as short 5th fingers bilaterally with clinodactyly of the 5th digit on the left hand. Review of the family history revealed a younger brother who was small for age but was proportionate. He was nondysmorphic and had no other clinical concerns. Considering that he has a normal karyotype and his parents are small in stature, no further cytogenetic investigations (e.g., array CGH) were performed.

**Figure 2:**
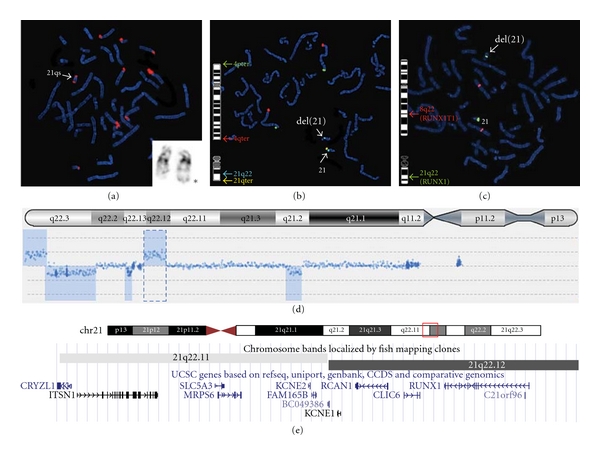
Complex rearrangement is revealed by array CGH. (a) FISH studies using acro-p probe targeting the short arms of all acrocentric chromosomes (red) were used to investigate the additional satellite on the long arm of 21qs. Insert shows the G-banded 21 chromosome with additional satellite on 21q (as indicated by ∗). (b) FISH studies using probes targeting 21q22 locus (encompassing *RUNX1* gene, aqua) and 21qter locus (yellow) were used to lineate the deleted chromosome 21. The del(21) lacks the yellow signal for the 21qter locus and has a diminished signal for the 21q22 locus. The 4pter and 4qter probes were used because they are in the same cocktail with the 21q22 and 21qter probes (Abbott Molecular Inc. ToTelVysion Vial no.4 probe set). (c) By using the *RUNX1*/*RUNX1T1* (previously known as *AML1*/*ETO*) dual color dual fusion translocation probe set (Abbott Molecular Inc), the FISH studies confirmed the diminished signal for the *RUNX1* locus. (d) Array CGH study detected a complex rearrangement on the long arm of chromosome 21 with 3 copy number gains and 2 copy number losses. (e) The second copy number variation (highlighted by the broken line in (d)) contains multiple annotated genes including *RUNX1*, as shown by the UCSC genome browser.

Cytogenetic investigations in the patient showed a chromosome abnormality with additional satellites on the distal long arm of one chromosome 21 (46,XY,21qs), which was confirmed by FISH studies using an Acro-p probe (Abbott Molecular Inc.) (Figure 2(a)). To determine whether this abnormality was familial, chromosome studies were performed on both parents, and the results were normal. Further FISH studies demonstrated a deletion of the 21q subtelomere region (Figure 2(b)) with a diminished signal for the *RUNX1* locus (Figure 2(c)), suggesting that one of the deletion breakpoints is located within the *RUNX1* locus. Due to the clinical significance of haploinsufficiency for the *RUNX1* gene (as discussed below), further investigation was undertaken to determine if the gene was completely deleted. Surprisingly, array CGH analysis using the Cytosure Syndrome Plus V2 2 × 105 k Oligonucleotide array platform (OGT) not only confirmed the complete deletion of the *RUNX1* gene but it also revealed a complex chromosomal rearrangement of chromosome 21 with interspersed duplications (×3) and deletions (×2) (Figure 2(d)). 

The first and most proximal copy number variation (CNV) is a gain which encompasses a region of 1.1 Mb from nucleotide 23,449,744 to 24,557,710. DGV revealed there are no reported benign CNVs fully encompassing this duplicated region. However, there are no known genes within this region (by UCSC genome browser), suggesting that this duplicated region is of no clinical significance. The second CNV is a loss which encompasses 1.81 Mb from nucleotide 34,965,815 to 36,781,907 and includes the gene *RUNX1* (Figure 2(e)). No reported benign CNVs have been found encompassing this loss (by DGV). Besides *RUNX1* which will be discussed later, there is another disease-associated OMIM gene *CLDN14*, which is associated with a type of autosomal recessive deafness. Since there is no concerns about the patient's hearing, haploinsufficiency of *CLDN14* has unknown clinical significance. The third CNV is a gain encompassing 0.42 Mb from nucleotide 37,847,805 to 38,276,148. There is no reported benign CNVs fully encompassing this gain (by DGV), and there is only one RefSeq gene *KCNJ6* located within it (by UCSC genome browser). Duplication of *KCNJ6* is of unknown clinical significance. The fourth CNV is also a gain of 4.1 Mb in size from nucleotide 40,917,977 to 45,022,823. Similar to the other two gains, there is no reported benign CNVs fully encompassing this region (by DGV). UCSC genome browser revealed several disease-associated OMIM genes (*TMPRSS3*, *CBS*, *CRYAA*, *CSTB*, *AIRE*, and* PFKL*). Mutations and/or deletions of these genes are associated with various diseases. However, extra copies of them are of unknown clinical significance. The fifth and most distal CNV is a terminal loss encompassing 1.82 Mb from nucleotide 45,096,184 to 46,920,235. DGV showed no reported benign CNVs fully encompassing this loss, and UCSC genome browser revealed several disease-associated OMIM genes (as follows). Biallelic loss-of-function mutations in *ITGB2*, *COL18A1*, *FTCD*, and *PCNT2* cause leukocyte adhesion deficiency (OMIM no. 116920), Knobloch syndrome type I (OMIM no. 267750), formiminotransferase deficiency (OMIM no. 229100), and type II icrocephalic osteodysplastic primordial dwarfism (OMIM no. 210720) respectively. Whether haploinsufficiency of these genes is associated with any clinical significance remains unknown. Mutations in *COL6A1* and *COL6A2* cause two autosomal dominant muscular diseases: Bethlem myopathy (OMIM no. 158810) and Ullrich congenital muscular dystrophy (OMIM no. 254090). Haploinsufficiency of the two genes could explain the 5th finger clinodactyly and the delay motor development observed in the patient [13, 14]. Of note, none of the gains encompass the Down syndrome critical regions. Besides, there is no probe between the fourth and the fifth CNVs and after the distal end of the fifth CNV.

*RUNX1*, deleted in the second CNV, is a hematopoietic transcription factor frequently involved in somatic or acquired chromosome translocations associated with leukemia [15, 16]. The constitutional mutations and deletions of *RUNX1* are known to be associated with familial platelet disorder with propensity to acute myelogenous leukemia (FPD/AML, OMIM no. 601399) [17, 18]. FPD/AML is an autosomal dominant disorder which is characterized by the prolonged bleeding time, mild-to-moderate thrombocytopenia with normal platelet size and morphology, with or without abnormal platelet aggregation in response to arachidonic acid [19–21]. Predisposition to the development of myeloid malignancies is another feature, with 20–50% progressing into AML and myelodysplasia. A lower cancer risk has been reported in individuals found to be haploinsufficient for *RUNX1* while partial deletions or missense mutations have been associated with a higher risk for hematologic malignancy. By using the array CGH analysis, we confirmed that the patient was haploinsufficient for *RUNX1* and thus has a decreased risk for AML and myelodysplasia. Furthermore, the complex rearrangement of chromosome 21 most likely represents *de novo* events. Therefore, the recurrence risk will be low for future pregnancies in this family.

### 3.3. Case No. 3

**Figure 3:**
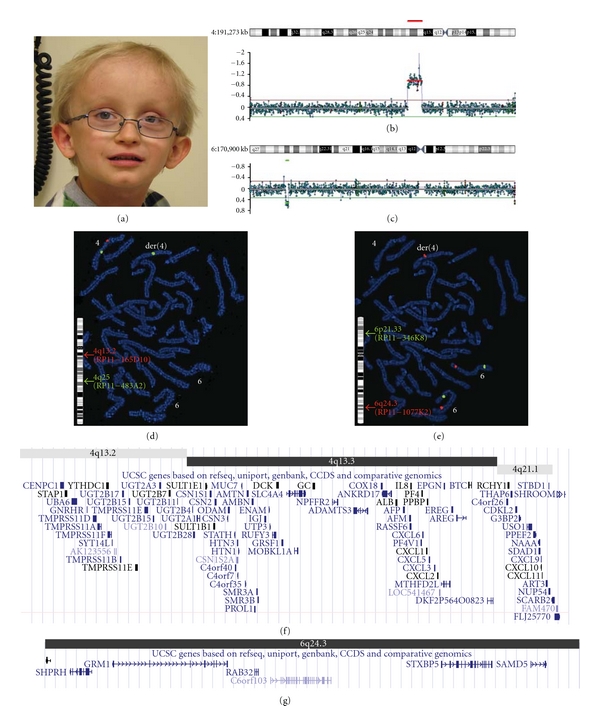
Array CGH identified a genomic loss and gain responsible for the patient's phenotype. (a) Patient's photo at the age of nine. His physical features demonstrate a relative macrocephaly with a prominent forehead, sparse hair and eyebrows, prominent ears which are low set, and cup-shaped, deep set eyes with esotropia, astigmatism, and possible congenital anomaly of the left optic disc. (b) Array CGH study detected a significant copy number loss on chromosome 4 (q13.2-q21.1). (c) Array CGH study also identified a significant copy number gain on chromosome 6 (q24.3). (d) The deletion was confirmed by FISH studies using a BAC probe RP11-165D10 (red) mapped to 4q13.2 and a control probe RP11-483A2 (green) mapped to 4q25. (e) The gain was confirmed by FISH studies using a BAC probe RP11-1077K2 (red) mapped to 6q24.3 and a control probe RP11-346K8 (green) mapped to 6p21.33. Of note, D and E are the same metaphase with different probe sets to visualize both chromosomes 4 and 6. The extra red signal appears to locate on the derivative chromosome 4. (f) Genes located within the deleted region 4q13.2-q21.1 (nucleotide 67,133,352 to 77,615,947, NCBI 36/HG 18) as shown by the UCSC genome browser. (g) Genes located within the duplicated region 6q24.3 (nucleotide 146,155,718 to 148,055,364, NCBI 36/HG 18) as shown by the UCSC genome browser.

This patient is the firstborn child to a healthy unrelated couple. At the time of birth the father and the mother's ages were 30 and 29, respectively. No anomalies were seen on the prenatal ultrasound, and the newborn was healthy. By nine months, the weight and length were at the 3rd percentile, while the head circumference remained at the 50th percentile. Developmental delay (delay in sitting, standing, and walking) was evident in the first year. At 16 months, severe expressive language delay was noted but receptive language was within normal limits. At 14 months, he had five seizures associated with fever and some focal signs. The patient was first seen at the genetics clinic at 20 months. No precise diagnosis had been made despite many investigations. The karyotype showed a normal male chromosome complement. Subtelomere FISH studies (TelVysion kit, Abbott Molecular Inc.) also showed a normal result. Other studies included UPD7, multiple biochemical tests, CT, brain MRI, skeletal survey, and thyroid studies which were all normal. At seven years nine months, the patient's height and weight were less then the 3rd percentile and head circumference remained at the 50th percentile. Now at the age of nine, he speaks intelligibly and in sentences. There are no behavior problems, no further seizures, and no sleep problems. He remains small despite a good appetite. His physical features demonstrate a relative macrocephaly with a prominent forehead, sparse hair and eyebrows, prominent ears which are low set, and cup-shaped, deep set eyes with esotropia, astigmatism, and possible congenital anomaly of the left optic disc (Figure 3(a)).

**Figure 4:**
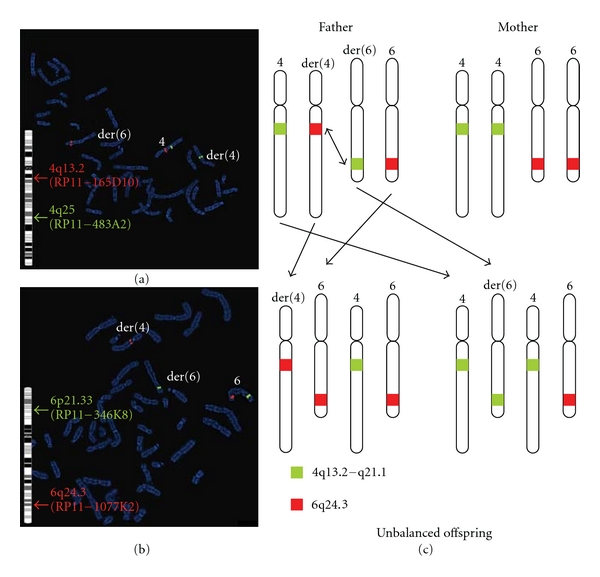
FISH studies revealed a four-break balanced complex rearrangement in the patient's father. (a) By using BAC probe RP11-165D10 (red) and control probe RP11-483A2 (green), FISH studies identified that the 4q13.2 segment has moved from derivative chromosome 4 to derivative chromosome 6. (b) By using BAC probe RP11-1077K2 (red) and control probe RP11-346K8 (green), FISH studies identified that the 6q24.3 segment has moved from derivative chromosome 6 to derivative chromosome 4. (c) A simplified diagram to show the increased risk of having an unbalanced offspring in this family. Green represents the 4q13.2-q21.1 segment and the red represents the 6q24.3 segments. In the father, the green and red segment, switch locations leading to unbalanced offspring with either 3 copies of the red segment and 1 copy of the green segment (as in the patient) or 3 copies of the green segment and 1 copy of the red segment.

Once the technology was developed locally, array CGH was performed on the peripheral blood of the patient using the CytoChip ISCA 8 × 60 K v2.0 Oligonucleotide array platform (BlueGnome). The array analysis identified two chromosomal abnormalities. The first one is a copy number loss of a 10.5 Mb region on the long arm of chromosome 4 (q13.2-q21.1) from nucleotide 67,133,352 to 77,615,947 (Figure 3(b)). According to DGV, there are no reported benign CNVs fully encompassing this large deletion. The clinical significance of the deletion will be discussed later. The second one is copy number gain of a 1.9 Mb region on the long arm of chromosome 6 (q24.3) from nucleotide 146,155,718 to 148,055,364 (NCBI 36/HG 18) (Figure 3(c)). DGV showed no reported benign CNVs fully encompassing this duplication, and UCSC genome browser revealed that there are no disease-associated OMIM genes within this region. In order to validate the array result, FISH studies were performed using a BAC probe RP11-135D10 mapping to the deleted region 4q13.2, a BAC probe RP11-1077K2 mapping to the duplicated region 6q24.3, two control probes on 4q25 (RP11-483A2) and on 6p21.33 (RP11-346K8) (RPC1-11 Human BAC library–CIHR Genome Resource Facility). Metaphase FISH confirmed the deletion at 4q13.2 and the duplication at 6q24.3. Interestingly, the third copy of 6q24.3 was located at 4q13 (Figures 3(d) and 3(e)). To further investigate whether this complex rearrangement is *de novo* or inherited, FISH studies were performed on the parental samples using the above 4 probes. The mother's result revealed a normal hybridization pattern with all probes hybridizing at the expected locations and with the expected copy number. The father's result, however, identified an abnormal hybridization pattern involving one chromosome 4 and one chromosome 6. The segment 4q13.2-q21.1 and the segment 6q24.3 are involved in a reciprocal insertional translocation which appears to be balanced (Figures 4(a) and 4(b)). Therefore, the patient's father has a four-break balanced complex rearrangement t(4;6)(q13.2q21.1;q24.3q24.3) which is not evident on the routine G-banded chromosomes. During the paternal meiosis, the der(4)t(4;6) and normal chromosome 6 segregated together and resulted in the chromosomal imbalances in this patient.

The patient's clinical presentation is most likely due to the deletion and duplication of many genes involved in the imbalanced regions (Figures 3(f) and 3(g)). However, there is a limited genotype-phenotype correlation in terms of each gene involved. The 10.5 Mb deletion on chromosome 4 contains 8 disease-associated OMIM genes (*GNRHR*, *MUC7*, *ENAM*, *SLC4A4*, *GC*, *ALB*, *AFP*, and *SCARB2*). Among these, *ENAM* mutations are associated with amelogenesis imperfecta, type 1B (OMIM no. 104500) which is an inherited defect of dental enamel formation with autosomal dominance as the likely mode of inheritance. However, this patient has a full set of primary teeth with normal tooth shape and enamel but delayed secondary teeth erupting. Currently, it is unclear whether haploinsufficiency of *ENAM* is responsible for the delay in secondary teeth erupting. The 1.9 Mb duplication on chromosome 6 does not contain any disease-associated OMIM genes (UCSC genome browser), and its clinical significance remains unknown.

Through combining array CGH and FISH techniques, we successfully identified the genomic imbalances responsible for the patient's clinical manifestation and further a familial chromosomal rearrangement which changes the predicted outcome and clinical management in this family. The father now carries a significant risk of having another abnormal liveborn with either der(4)t(4;6) as in the patient or der(6)t(4;6) (Figure 4(c)). Therefore, prenatal diagnosis will be offered for any future pregnancies. The patient has two siblings who are both normal. Cytogenetic testing will be available to determine the carrier status when they become adults. 

## 4. Conclusions 

The wide application of array CGH has remarkably improved the detection of DNA copy number variation and complex chromosome rearrangement. It has revolutionized the diagnostic process of patients with global developmental delay, dysmorphic features, multiple congenital anomalies, as well as complicated pregnancy at risk for chromosomal aberrations. By identifying the genomic imbalances responsible for the clinical presentation in patients, clinicians can provide an accurate diagnosis, predict the potential risk in the future and alter the clinical management in the patients and/or their families.
